# Imobilization of trypsin in films of PVA/CGP

**DOI:** 10.1186/1753-6561-8-S4-P217

**Published:** 2014-10-01

**Authors:** Bruna Moreira, Karla Batista, Kátia Fernandes

**Affiliations:** 1 Departamento de Bioquímica e Biologia Molecular, Laboratório de Química de Proteínas, Universidade Federal de Goiás, Cx. Postal 131, 74001-970, Goiânia, GO, Brazil

## Background

Trypsin is an enzyme widely used for digestion of peptides in proteomic research. However, this enzyme is one of the least stable of the neutral proteases and presents rapid autolysis, which causes a decrease in catalytic efficiency and a consequential increase in costs of biotechnological process. Immobilization of the enzyme offers the advantages of enhanced stability, protection against autolysis and the possibility of repeated use of the catalytic material. In addition, the preparation of a successfully immobilized enzyme depends very much on the method of immobilization employed and on the chemical characteristics of the support [1,2]. In this sense, a biodegradable film produced by blending polyvinyl alcohol (PVA) and cashew gum polysaccharides (CGP) was tested as support for trypsin immobilization.

## Methods

The blends used in this sense were produced using the methodology described by Silva et.al (2013) [3]. The method chooses for immobilization was covalent linking and the hydrolytic activity of PVA/CGP/Trypsin film (cm2) was assayed using benzoil-DL-arginina-p-nitroanilida (BapNa) as substrate. The results were analyzed using the software *Statistica 7.0 *(StatSoft Inc., Tulsa, OK, USA).One unit (UE) of enzyme activity was defined as the amount of enzyme that produces increase of 0, 1 on absorbance. The PVA/CGP/Trypsin was tested repeatedly for hydrolysis of BapNa, because this parameter it is important in continuous use reactor for bioconversion processes. For this the PVA/CGP /Trypsin films were stored in different conditions e tested your activity at each week.

## Results and conclusions

Multivariate analysis results showed that all factors significantly (P < 0.05) affected the activity, and that factor interaction between time and temperature had no effect (P > 0.05) on this response. The linear factors time and temperature positively affected response, and the linear term pH negatively affected the activity. The response surface showed a maximum region corresponding to at 25º C, pH 4, 0 and 60 minutes corresponding to 4,79UE.

The regression analysis showed an adequate fit (r^2 ^= 0, 95) of experimental values to the second-order polynomial model as a function of significant factors. The mathematical model is represented in the following equation:

$$\text{Y}=-0,11+0,046\;{\text{X}}_{1}+0,004\;{\text{X}}_{2}+0,0002\;{{\text{X}}^{2}}_{2}+0,003\;{\text{X}}_{3}+0,0001\;{{\text{X}}^{2}}_{3}-0,002{\text{X}}_{1}{\text{X}}_{2}+0,001\;{\text{X}}_{1}{\text{X}}_{3}-0,0001\;{\text{X}}_{2}{\text{X}}_{3},$$

where, Y, X_1_, X_2 _and X_3 _denoted absorbance at 405 nm, pH, time and temperature, respectively. The high operational stability of PVA/Chitosan/Trypsin film was a characteristic that indicates in this sense, showing stable for about three weeks at different storage conditions, leading to the believe that the immobilization process gives a better stability for the enzyme reaction when compared to the free enzyme.
